# Ag Nanoparticle-Decorated MoS_2_ Nanosheets for Enhancing Electrochemical Performance in Lithium Storage

**DOI:** 10.3390/nano11030626

**Published:** 2021-03-03

**Authors:** Thang Phan Nguyen, Il Tae Kim

**Affiliations:** Department of Chemical and Biological Engineering, Gachon University, Seongnam-si 13120, Gyeonggi-do, Korea; phanthang87@gmail.com

**Keywords:** MoS_2_, Ag, nanosheets, nanoparticle, lithium-ion battery, high rate

## Abstract

Metallic phase 1T MoS_2_ is a well-known potential anode for enhancing the electrochemical performance of lithium-ion batteries owing to its mechanical/chemical stability and high conductivity. However, during the lithiation/delithiation process, MoS_2_ nanosheets (NSs) tend to restack to form bulky structures that deteriorate the cycling performance of bare MoS_2_ anodes. In this study, we prepared Ag nanoparticle (NP)-decorated 1T MoS_2_ NSs via a liquid exfoliation method with lithium intercalation and simple reduction of AgNO_3_ in NaBH_4_. Ag NPs were uniformly distributed on the MoS_2_ surface with the assistance of 3-mercapto propionic acid. Ag NPs with the size of a few nanometers enhanced the conductivity of the MoS_2_ NS and improved the electrochemical performance of the MoS_2_ anode. Specifically, the anode designated as Ag3@MoS_2_ (prepared with AgNO_3_ and MoS_2_ in a weight ratio of 1:10) exhibited the best cycling performance and delivered a reversible specific capacity of 510 mAh·g^−1^ (approximately 73% of the initial capacity) after 100 cycles. Moreover, the rate performance of this sample had a remarkable recovery capacity of ~100% when the current decreased from 1 to 0.1 A·g^−1^. The results indicate that the Ag nanoparticle-decorated 1T MoS_2_ can be employed as a high-rate capacity anode in lithium-ion storage applications.

## 1. Introduction

Recently, there has been increasing interest among researchers in transition metal chalcogenides (TMCs), which are graphene-like two-dimensional (2D) materials consisting of a transition metal atom layer sandwiched between two chalcogenide atom layers. Each monolayer of TMC is formed as a 2D structured layer, and these layers are bonded to each other by van der Waals forces in the bulk structure. Therefore, the TMCs can be easily exfoliated into a single layer or a few layers. Moreover, various properties of these 2D materials have been investigated, and they have been found to be superior to bulk materials in strength, physical and chemical stability, and conductivity [1,2,3,4]. Therefore, TMCs have been employed in several electronic, optical, and energy conversion/storage applications, for example, in energy applications such as solar cells, light emitting diodes, hydrogen evolution reactions, and metal-ion batteries [5,6,7,8,9,10,11,12]. Among TMC materials, MoS_2_ shows great potential for easy processing and high stability. Therefore, numerous studies have reported on its characteristics and applications. In particular, with a direct band-gap structure, the 1T phase of MoS_2_ nanosheets (NSs) is attractive owing to its high mechanical/chemical stability and high conductivity [13,14,15,16]. Recently, MoS_2_ NSs have been used as potential candidates for anodes in lithium-ion batteries (LIBs) [17,18,19,20,21,22]. With a nanosheet structure, MoS_2_ has a large surface area and flexibility for the lithiation and delithiation processes and enhances the electrochemical performance of LIBs. Furthermore, the 1T phase of MoS_2_, with metallic properties, can afford high conductivity, which facilitates the processes of lithiation and delithiation. Various methods have been used to enhance the electrochemical performance of MoS_2_ in LIBs by using metals doping, metal particles or metal oxides. Zhu et al. used TiO_2_ nanoparticles (NPs) decorating on 2H- MoS_2_ NS via hydrothermal method to achieve the reversible capacity of 604 mAh·g^−1^ after 100 cycles [23]. Pan et al. developed Ag methanesulfonic-acid capped NPs with 2H-MoS_2_ NSs by sonication method to get high reversible capacity of ~920 mAh·g^−1^ after 50 cycles [24]. In addition, Li et al. synthesized lithium molten salt of MoS_2_ as a precursor at 1050 °C for liquid exfoliation of 1T MoS_2_ [25]. This report showed that the superior properties of 1T MoS_2_ to 2H MoS_2_ due to the existence of abundant monolayer structures, providing diffusion path for lithium ion insertion/desertion. Wang et al. reported vertically aligned MoS_2_ NSs patterned on graphene for LIBs, which exhibited high-rate energy storage [26]. This structure also enables sodium-ion storage capability. Tang et al. developed hollow 1T MoS_2_ grown on carbon cloth and demonstrated high rate performance, high capacity, and good stability in sodium-ion batteries [27]. Li et al. combined 1T MoS_2_ with MnO in lithium molten salts assisted with a ball milling method to develop a high-stability LIB anode. This MoS_2_/MnO composite anode retained a high capacity of ~589 mAh·g^−1^ after 2000 cycles [28]. Bai et al. fabricated a 1T MoS_2_/C hybrid anode material through a hydrothermal method [20]. These carbon-covered MoS_2_ NS materials also exhibited a high rate performance in LIBs. Therefore, 1T phase MoS_2_ could be a potential anode for high capacity and high rate performance in LIBs. However, the commercialization of MoS_2_ anode materials requires an easy fabrication process and further improvement in stability and rate performance.

In this study, we successfully investigated Ag NP-decorated 1T MoS_2_ nanosheets as a potential anode for high-rate performance and stable LIBs. MoS_2_ was prepared by a liquid chemical exfoliation method with lithium intercalation. By adding 3-mercapto propionic acid (MPA) as a functional group, Ag NPs were uniformly decorated on the MoS_2_ surface. The presence of Ag NPs not only improves the specific capacity but also significantly enhances the rate performance and stability of the anode material in lithium storage. Notably, the Ag3@MoS_2_ anode can restore ~100% capacity after high-rate cycling.

## 2. Materials and Methods

### 2.1. Chemical Materials

Molybdenum (IV) sulfide (MoS_2_, powder), silver nitrate (AgNO_3_, 99%), MPA (99%), polyvinylidene fluoride (PVDF, MW 534,000), *N*-methyl-2-pyrrolidinone (NVP, anhydrous, 99.5%), and a 2.5 M solution of n-butyllithium ion hexane and sodium borohydride (NaBH_4_, 99%) were purchased from Sigma-Aldrich Inc. (St. Louis, MO, USA). Super P amorphous carbon black (C, approximately 40 nm, 99.99%) was purchased from Alpha Aesar Inc. (Haverhill, MA, USA).

### 2.2. Synthesis MoS_2_ NSs

MoS_2_ NSs were prepared as described in our previous report [17]. The loading of butyllithium was conducted in Ar-filled glovebox to prevent the reactions between butyllithium and oxygen/moister. First, 1.0 g of MoS_2_ powder was added to 3 mL of 2.5 M butyllithium in hexane. Then, the solution was sealed with parafilm, taken out to be sonicated for 1 h, and kept for two days to obtain lithium-intercalated MoS_2_ (Li_x_MoS_2_) in glove box. The excess lithium was removed by washing with hexane. The obtained Li_x_MoS_2_ was placed in 100 mL of deionized (DI) water. The interlayer lithium reacted with DI water to break the layer structure of the bulk MoS_2_ and form MoS_2_ NSs. The solution was further sonicated for 1 h and stirred for 1 h to obtain a complete dispersion of the MoS_2_ NS. Finally, the dispersion was centrifuged and washed four times to remove excess lithium.

### 2.3. Ag-Decorated MoS_2_ NS

The surface of the NSs was modified by MPA in order to obtain Ag-decorated MoS_2_ NSs. The prepared LixMoS_2_, after being washed with hexane, was added to 100 mL of 0.045 M MPA solution. This process was similar to the synthesis of MoS_2_ NSs. After sonication and washing with DI water, MPA-modified MoS_2_ NSs were redispersed in DI water via sonication. Then, amounts of AgNO_3_ with different weight ratios (1:50, 1: 20, and 1:10) to MoS_2_ were added to the solution during stirring, and the samples were denoted as Ag1@MoS_2_, Ag2@MoS_2_, and Ag3@MoS_2_, respectively. Naturally, MPA contains both thiol and carboxyl groups. The thiol group is able to exchange with the S atom on MoS_2_ with the appearance of Li ions [29,30]. Meanwhile, the carboxyl group induces a partial negative charge, which attracts Ag^+^ ions in the solution. Then, a solution of 0.5 M NaBH_4_ was added to the aforementioned solution to reduce Ag^+^ to Ag nanoparticles. The solution was further washed via centrifugation with DI water three times to remove NaBO_2_. The final product was obtained after drying at 70 °C for 12 h.

### 2.4. Characterization

Scanning electron microscopy (SEM; Hitachi S4700, Tokyo, Japan) and transmission electron microscopy (TEM; TECNAI G2F30, FEI Corp., Hillsboro, OR, USA) were used to analyze the morphologies and sizes of the as-prepared materials. Samples were coated a few-nanometers Pt layer via magnetron sputtering system for high quality SEM images. A high-resolution X-ray diffractometer (XRD; SmartLab, Rigaku, Tokyo, Japan) was used to investigate material structures. XRD patterns were recorded over the 2θ range 10–70°.

### 2.5. Electrochemical Measurements

Anode materials were assembled in a half-cell LIB using coin-type cells (CR 2032, Rotech Inc., Gwangju, Korea). Typically, the anode electrode was prepared using a doctor blade on a Cu foil using a slurry of 70% active material, 15% PVDF, and 15% Super P in NVP. Then, the electrodes were dried in a vacuum oven at 70 °C for 24 h before use. The anodes were punched into 12 mm diameter circular disks. The loading of active materials was ~0.7–1.0 mg cm^−2^. Then, battery half-cell structures were assembled in an Ar-filled glovebox with positive pressure (>1.0 atm). Lithium foil and polyethylene were used as the reference electrode and separator, respectively. A solution of 1 M LiPF_6_ in ethylene carbonate-diethylene carbonate (1:1 by volume) was employed as the electrolyte. Galvanostatic electrochemical discharge/charge analysis of the different cells was performed using a battery cycle tester (WBCS3000, WonAtech Co., Ltd., Seocho-gu, Seoul, Korea) over the voltage range 0.01–3.00 V versus Li/Li+. Electrochemical impedance spectroscopy (EIS) and cyclic voltammetry (CV) tests were performed using a ZIVE MP1 apparatus (WonAtech Co., Ltd., Seocho-gu, Seoul, Korea). EIS measurements were recorded at 3.0 V over the frequency range between 100 kHz and 0.1 Hz. CV tests were performed over the voltage range 0.01–3.0 V at a scan rate of 0.1 mV·s^−1^.

## 3. Results and Discussion

The MoS_2_ NSs were fabricated using a liquid exfoliation method [17]. MoS_2_ bulk powder was mixed with butyllithium in hexane to form intercalated lithium ions in MoS_2_ as interlayer structures. The lithium ions easily filled the MoS_2_, forming inter-layers between two MoS_2_ layers by the following process:

(1) $$MoS_{2}+xLi\;\to \;Li_{x}MoS_{2}\;$$

According to Dines [31], the x value is in the range 1.1–1.5. This implies that the lithium ions easily fill the bulk MoS_2_. Then, reactions between the intercalated lithium and water create hydroxide ions and hydrogen gas. This reaction and the generated gas exfoliate the MoS_2_ layers. Finally, the bulk MoS_2_ is cleaved into MoS_2_ NSs.

**Figure 1:**
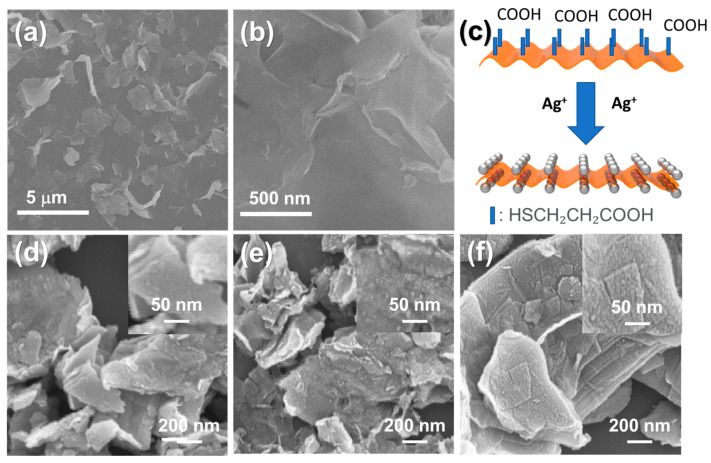
(**a**,**b**) Scanning electron microscopy (SEM) images of MoS_2_ nanosheets (NSs); (**c**) illustration of Ag-decorated MoS_2_ NS; SEM images of (**d**) Ag1@MoS_2_, (**e**) Ag2@MoS_2_, and (**f**) Ag3@MoS_2_ NSs.

Figure 1a,b are SEM images of exfoliated MoS_2_ NSs at different scale bars of 5 μm and 500 nm, respectively. The lateral size of the MoS_2_ NSs was between 100 nm and 3 μm. This wide range of MoS_2_ sizes is because of the strong reaction with Li(1) and the random shape of the bulk MoS_2_ powder. According to previous reports, the butyllithium intercalation process converts the 2H MoS_2_ to 1T due to the effect of lithium ion [10,32]. In this phase, the material could have high conductivity (10–100 S cm^−1^), which is about 10^5^ times that of 2H-MoS_2_ [25,33]. Therefore, it is thought that highly conductive 1T MoS_2_ phase would generate different electrochemical performance (discussed later). Besides, AgNO_3_ was selected as the Ag source in order to decorate Ag nanoparticles on the MoS_2_. MoS_2_ NSs were functionalized by MPA to prevent random decoration and achieve uniform decoration. Using this functional group, metal ions are easily attracted to the partial negative charge of -COO^−^ to uniformly distribute the Ag^+^ ions. Then, H_2_ generated by the hydrolysis of NaBH_4_ reduces Ag^+^ ions into Ag NPs, as illustrated in Figure 1c. Three samples with different AgNO_3_:MoS_2_ weight ratios—1:50, 1:20, and 1:10—were prepared, and, as aforementioned, they are designated herein as Ag1@MoS_2_, Ag2@MoS_2_, and Ag3@MoS_2_, respectively. Figure 1d, showing the Ag1@MoS_2_ sample and an inset image of its magnified surface, indicates that the surface of MoS_2_ has only a few Ag NPs owing to the small amount of Ag^+^ used. When the amount of AgNO_3_ was increased in Ag2@MoS_2_, the Ag NPs were more uniformly decorated with higher density. Figure 1e and its inset image show uniform Ag NPs on the MoS_2_ surface; however, they do not fully cover the surface. The 1:10 ratio of the Ag3@MoS_2_ sample reveals the highest coverage of Ag nanoparticles with a size of <10 nm on the MoS_2_ nanosheets, as shown in Figure 1f.

**Figure 2:**
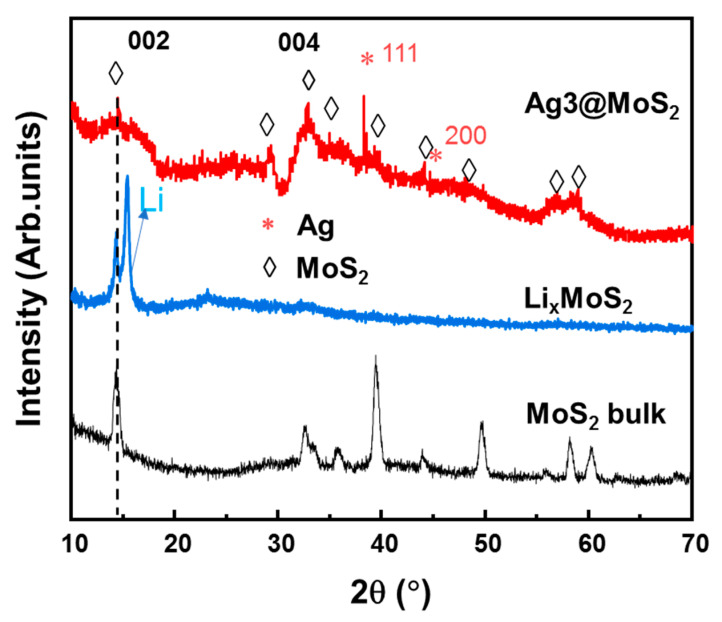
X-ray diffraction patterns of bulk MoS_2_, Li_x_MoS_2_, and Ag3@MoS_2_ materials.

The structure of MoS_2_ and Ag-decorated MoS_2_ were analyzed by XRD. Figure 2 shows the XRD patterns of bulk MoS_2_, Li_x_MoS_2_, and Ag-decorated MoS_2_ NSs. It is noted that the bulk MoS_2_ powder contains many peaks contributed by various planes in the lattice. However, after lithium ions are intercalated in the MoS_2_ layers, the XRD pattern of Li_x_MoS_2_ has a main peak for the (002) plane of MoS_2_ at ~14.36° and a peak at 15.1°, which is related to the interlayer plane of Li between the MoS_2_ layers. The other peaks were much reduced and negligible due to the high intensity of (002) plane. After exfoliation and Ag decoration, the sample shows some main peaks for MoS_2_ at ~14.4°, ~29.0°, and 32.7°, which correspond to the (002), (004), and (100) planes according to the #JCPDS card number 00-037-1492. The XRD peaks of MoS_2_ are broadened in comparison to those of the bulk material. According to the Scherrer equation, the average size of the crystals can be calculated by D = 0.9λ/βcosθ, where D is the average size, λ is the X-ray wavelength, β is the full width at half maximum of the peak, and θ is the diffraction angle. Thus, the broadening of these peaks indicates small crystal sizes in those planes, which implies that the bulk MoS_2_ material was exfoliated into nanosheets. These results are similar to other synthesized MoS_2_ nanosheets by bottom-up methods such as hydrothermal method and hot injection method [34,35,36,37]. The appearance of Ag peaks for the (111) and (200) planes confirms the successful reduction of AgNO_3_ into Ag nanoparticles.

**Figure 3:**
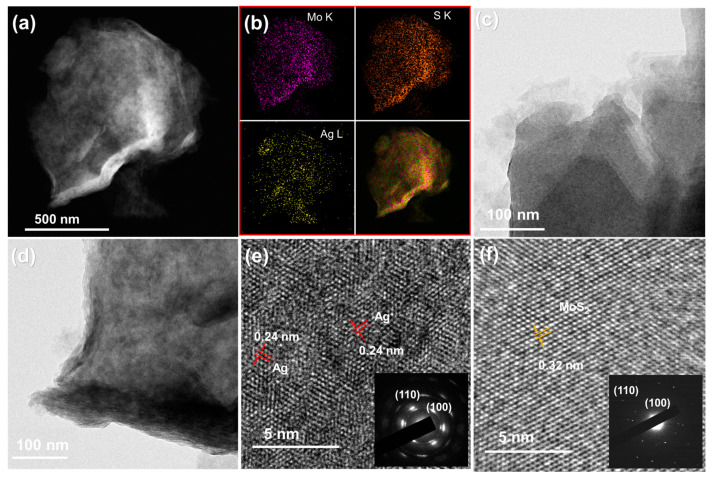
(**a**,**b**) Transmission electron microscopy (TEM) image and element mapping images of Ag3@MoS_2_ materials; (**c**,**f**) TEM, high-resolution TEM (HRTEM) with inset selected area electron diffraction (SAED) pattern of MoS_2_ NS; (**d**,**e**) TEM, HRTEM with inset SAED pattern of Ag3@MoS_2_ materials.

The structure of Ag-decorated MoS_2_ was further confirmed by TEM measurements. Figure 3a,b shows TEM images with individual and overlaid elemental mapping images of Mo (K), S (K), and Ag (L) elements. The Mo and S atoms are clearly shown with a high density of purple and orange colors, indicating the formation of MoS_2_ NSs. Ag atoms are uniformly distributed in the MoS_2_ area, confirming the successful decoration of Ag nanoparticles on MoS_2_. Figure 3c,f show TEM images and high-resolution TEM (HRTEM) images with an inset of the selected area electron diffraction (SAED) pattern of the MoS_2_ NSs. The MoS_2_ NS surface shows a clear lattice spacing distance of 0.32 nm, which corresponds to the (004) plane. Figure 3d,e shows TEM and HRTEM images of Ag3@MoS_2_ with an inset figure. The TEM image has many dark points representing Ag particles decorated on MoS_2_. The HRTEM image was also analyzed to reveal the lattice spacing of 0.24 nm resulting from the Ag lattice structure. The size of the Ag NPs is a few nanometers. The SAED pattern of the Ag-decorated sample is changed in comparison to that of the pure MoS_2_ sample. The pattern shows that the reflective planes reveal wide, blurred points, which could be because of the overlap of Ag NPs on the MoS_2_ NSs. The high uniformity of Ag decoration on MoS_2_ NSs is, thus, confirmed.

**Figure 4:**
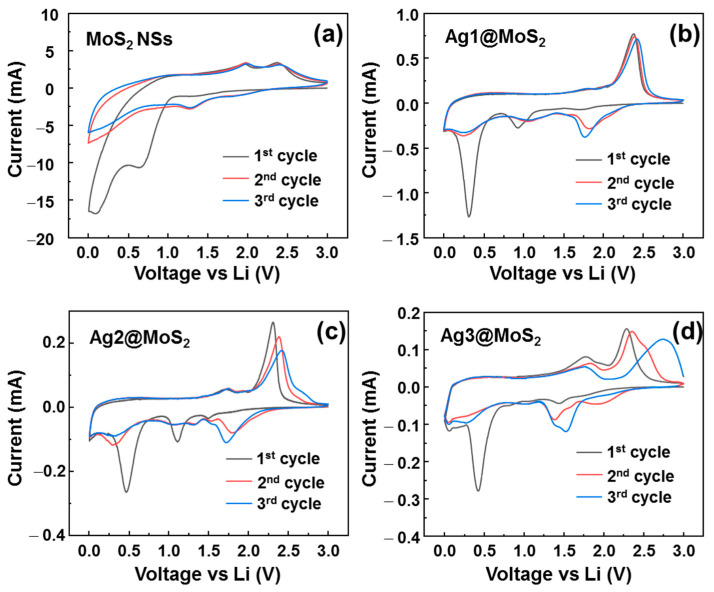
Cyclic voltammetry (CV) profiles of (**a**) MoS_2_ NSs, (**b**) Ag1@MoS_2_, (**c**) Ag2@MoS_2_ and (**d**) Ag3@MoS_2_ anodes, over the initial three cycles.

To investigate the effect of Ag-decorated MoS_2_ NSs on the electrochemical properties in lithium batteries, CV tests were performed at a scan rate of 0.1 mV·s^−1^ over the range 0.01–3.00 V (vs. Li/Li^+^). Figure 4a–d shows the initial three CV curves for MoS_2_ NSs and Ag1/2/3@MoS_2_ anodes. The electrochemical processes in the anode can be expressed by the following reactions:

(2) $$MoS_{2}+xLi^{+}+xe^{-}\to Li_{x}MoS_{2}$$

(3) $$Li_{x}MoS_{2}\;\to Li_{2}S+Mo$$

(4) $$Li^{+}\;+\;e^{-}\;+\;electrolyte\;\to SEI\;layer$$

(5) $$Ag+yLi^{+}+ye^{-}\to Li_{y}Ag$$

In the cathodic process, the MoS_2_ NSs anode shows a peak at 1.3 V (vs. Li/Li^+^), which is the intercalation process of lithium ions into MoS_2_ NSs to form Li_x_MoS_2_, corresponding to reaction (2) [26]. The strong peak at 0.5–1.0 V (vs. Li/Li^+^) is related to the strong formation of a solid electrolyte interface (SEI) layer and the transformation of Li_x_MoS_2_ to metallic Mo nanoparticles and the Li_2_S conversion reaction, as shown in Equations (3) and (4) [17,19]. In the 2nd and 3rd cycles, the SEI layer is stable; therefore, the peak at 0.5–1.0 V is significantly reduced. The peak at 1.3 V is strong and stable, indicating stable lithium intercalation process. In the anodic process, the peaks at 1.8 and 2.3 V are associated with the oxidation of Mo to Mo^+4^ and the delithiation of Li_2_S to sulfur, respectively. In the case of the Ag1@MoS_2_ anode, the CV curves indicate some different electrochemical processes. The intercalation peak in the first cycle is shifted to ~1.0 V (vs. Li/Li^+^). The CV profiles mainly have two pairs of redox peaks at 1.1/1.7 V and 1.75/2.3 V, which are related to the multiple steps of lithiation and delithiation. When the amount of Ag is increased, the reduction peaks shift to higher potentials. In the Ag2@MoS_2_ anode, there are two pairs of redox peaks at 1.3/1.75 and 1.75/2.4 V. The peak at ~0.3 V is the formation of Ag–Li alloys [38]. The dealloying peak of Ag–Li is not shown as a clear peak; however, the hump at 0.2–0.5 V may indicate the multiple Ag–Li phases of the de-alloying process [38]. Notably, when the Ag content is increased, the oxidation peak position shifts to a higher potential, and the shape is broadened. This might be because of the enhanced multiple steps of the oxidation process for Li_2_S [39].

**Figure 5:**
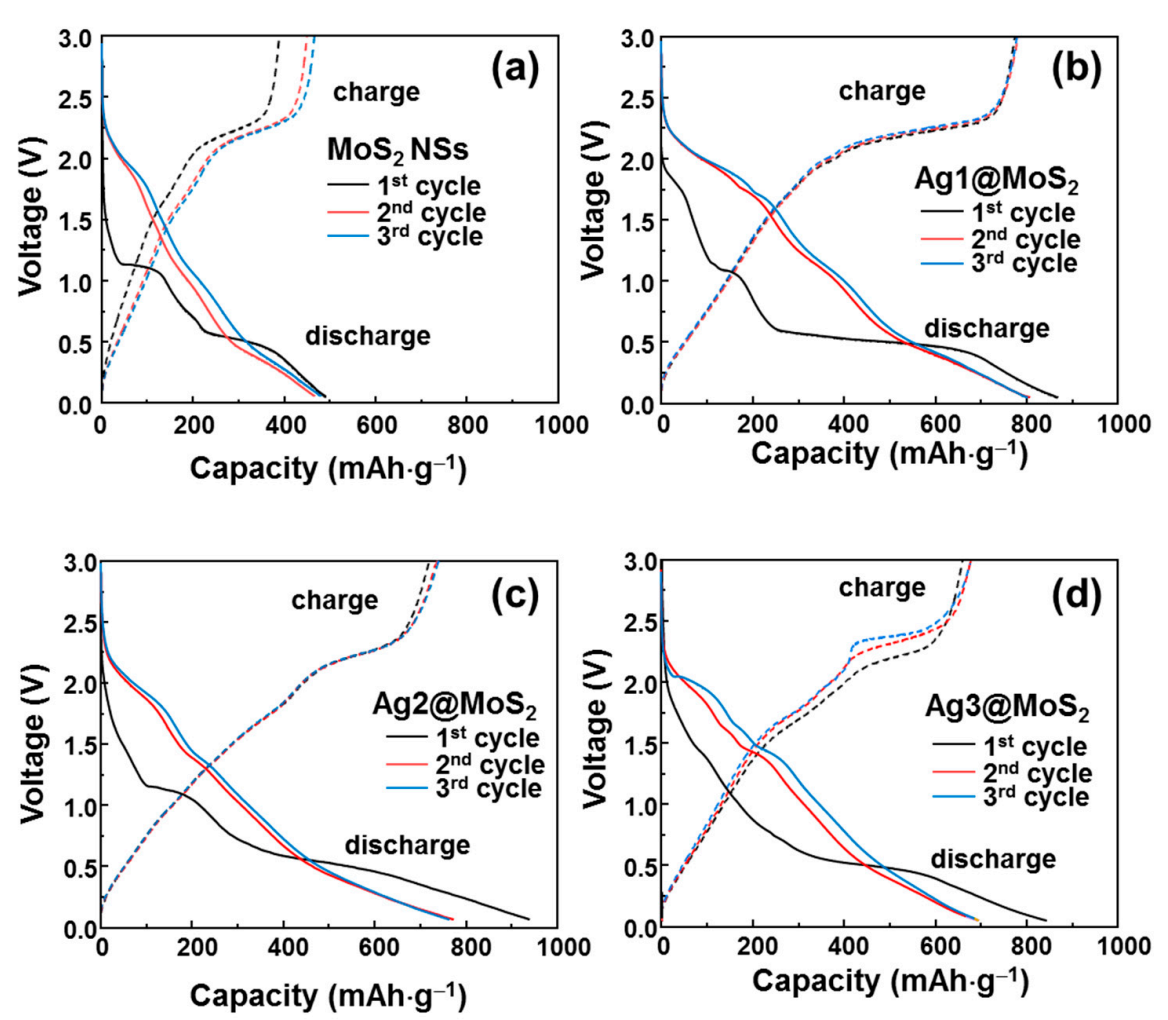
Initial voltage profiles of (**a**) MoS_2_ NSs, (**b**) Ag1@MoS_2_, (**c**) Ag2@MoS_2,_ and (**d**) Ag3@MoS_2_ anodes.

The initial voltage profiles of the as-prepared anodes are shown in Figure 5. It can be observed that with Ag decoration, the discharge/charge capacity of the MoS_2_ anodes is improved from ~500 to ~900 mAh·g^−1^, while the MoS_2_ NS reveals a low initial discharge/charge capacity of only ~490/466 mAh·g^−1^. Notably, the capacity does not decrease significantly during the initial cycles. The Ag1@MoS_2_ demonstrates a high initial discharge/charge capacity of 866/780 mAh·g^−1^ and ~800/773 mAh·g^−1^ at the 2nd and 3rd cycles, which is >92% of that of the first cycle. The Ag2@MoS_2_ anode exhibits a high discharge/charge capacity of 937/720 mAh·g^−1^ at the 1^st^ cycle and ~773/739 mAh·g^−1^ at the 2nd and 3rd cycles. The enhancement of the lithium storage capacity could be owing to the Ag decoration improving the conductivity of the materials, thus, facilitating the lithiation/delithiation process. For the Ag3@MoS_2_ anode, the discharge/charge capacity is slightly reduced to ~840/660 at the 1^st^ cycle and 694/679 mAh·g^−1^ at the 2nd and 3rd cycles, which can be attributed to the higher amount of Ag NPs, leading to stable cyclic performance. Meanwhile, the initial voltage profile of the MoS_2_ anode shows a sloping plateau at ~1.8 V, which is ascribed to the insertion of Li ions into MoS_2_, according to Equation (2) [26]. A sloping plateau at ~1.2 V corresponds to the reaction of lithium with sulfur in Equation (4). At below 0.5 V, the sloping plateau is related to the deep conversion reaction of lithium with MoS_2_ and formation of the SEI layer. The MoS_2_ NSs, Ag1@MoS_2_, and Ag2@MoS_2_ have similar reaction potentials. In contrast, the Ag3@MoS_2_ electrode has a higher plateau voltage at 2 V. The charging process of the Ag3@MoS_2_ anode also shows a higher plateau at ~2.4 V, higher than the plateau at ~2.2 V for the other electrodes. This process is because of a shift in the redox potential, which was indicated by the CV profiles of these anodes.

**Figure 6:**
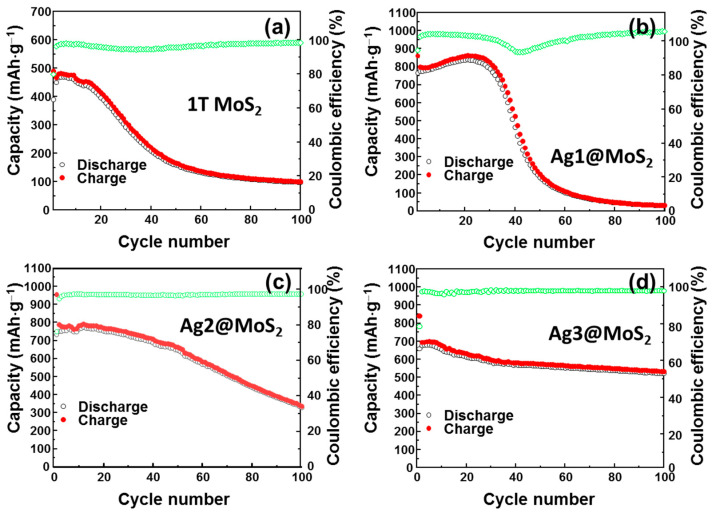
Cyclic performance of (**a**) MoS_2_ NSs, (**b**) Ag1@MoS_2_, (**c**) Ag2@MoS_2_, and (**d**) Ag3@MoS_2_ anodes.

To further investigate the long-term cyclability, the performance of these anodes was analyzed in half-cells for 100 charge-discharge cycles at a current rate of 100 mA·g^−1^, as shown in Figure 6a–d. The MoS_2_ NS anode exhibits a stable cycling performance during the initial 15 cycles; however, the capacity gradually decays thereafter and remarkably fades from the 30th cycle to the 100th cycle to a capacity of ~100 mAh·g^−1^. This can be attributed to the formation of a broken MoS_2_ structure after the cycling process and a restacking of the MoS_2_ NS layers. By addition of a small amount of Ag nanoparticles, the Ag1@MoS_2_ anode improves the electrochemical performance for the initial 30 cycles; however, a dramatic capacity decay still occurs after that. A small amount of Ag enhances the conductivity of MoS_2_; however, the applied amount in the Ag1@MoS_2_ anode does not appear to be enough to protect the entire MoS_2_ structure. Thus, after 40 cycles, the MoS_2_ NS structure collapses, and the lithium storage capability worsens. A further increase in the amount of Ag diminishes the collapse of the MoS_2_ structure, leading to an enhancement in the cycling performance of the anode. The Ag2@MoS_2_ anode retains a capacity of 330 mAh·g^−1^ up to 100 cycles. The Ag3@MoS_2_ anode exhibits the best cycling performance; it retains a specific capacity of ~510 mAh·g^−1^ after 100 cycles, corresponding to a capacity retention of ~73%.

**Figure 7:**
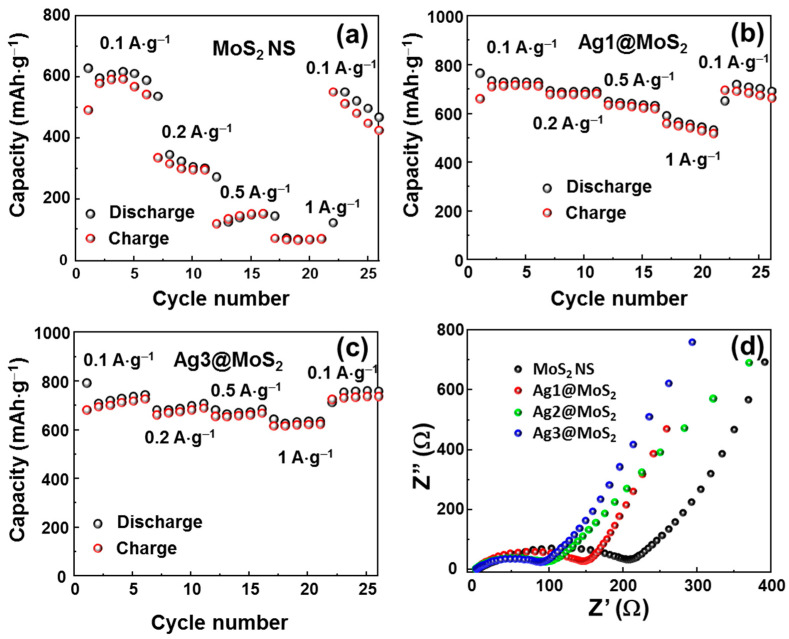
Rate performance of (**a**) MoS_2_ NSs, (**b**) Ag1@MoS_2_, (**c**) Ag3@MoS_2_, and (**d**) Nyquist plots of MoS_2_ without/with Ag-decorated anodes.

The discharge/charge rate performance of MoS_2_ NS without and with Ag nanoparticle decoration is shown in Figure 7a–c. These cycles were recorded at 0.1, 0.2, 0.5, and 1.0 A·g^−1^. As observed, bare MoS_2_ NSs show an inferior rate performance with a dramatic decrease in capacity from ~600 to 300, 180, and ~100 mAh·g^−1^, which corresponds to capacity retentions of 50, 30, and 17%, respectively. The restored capacity when going back from 1 to 0.1 A·g^−1^ is ~430 mAh·g^−1^, which is approximately 72%. When Ag nanoparticles are introduced, however, the rate performance of the anodes significantly improves. The Ag1@MoS_2_ anode shows capacity reductions from ~730 to 690, 650, and 550 mAh·g^−1^, respectively, which correspond to capacity retentions of 94, 89, and 75%, respectively. The restored capacity reaches ~676 mAh·g^−1^ (~92% of the initial capacity). Moreover, the Ag3@MoS_2_ electrode demonstrates the best rate performance, exhibiting capacity retentions of 98%, 96%, and 92%, which correspond to capacity values from 700 to 690, 677, and 646 mAh·g^−1^, respectively. The capacity after the high-rate test at 1 A·g^−1^ was 100% recovered when returning to 0.1 A·g^−1^. To further investigate the effect of Ag in MoS_2_, EIS measurements were performed to evaluate the change in the charge-transfer resistance, as illustrated in Figure 7d. The equivalent circuit using the modified Randles model contains a series resistance, SEI resistance, charge-transfer resistance, and a Warburg impedance element, and this was used to simulate the Nyquist plot [40]. The extracted charge-transfer resistances of the MoS_2_ NS and Ag1/2/3@MoS_2_ anodes are 210.5, 152.3, 99.1, and 95.8 Ω, respectively. The presence of Ag NPs clearly leads to an improvement in the anode conductivity [41,42]. Between the Ag2@MoS_2_ and the Ag3@MoS_2_ anode, the charge-transfer resistance is not significantly reduced, which indicates that the amount of Ag NPs is sufficient to decorate and enhance the electronic properties of the MoS_2_ NSs. Thus, the 1:10 weight ratio of AgNO_3_:MoS_2_ can contribute to the best performance in lithium-ion storage of MoS_2_ anode materials.

Recent works are summarized in Table 1. The reversible capacity of modified MoS_2_ NSs can deliver up to ~1000 mAh·g^−1^. From our method, the MoS_2_ bulk was exfoliated into few layer MoS_2_, with decoration of Ag on MoS_2_ NSs. The Ag decorated MoS_2_ NSs exhibited stable cyclability and high-rate performance. Moreover, the butyllithium assisted technique and uniform decorating technique for metal-particles can be easily scaled up to industrial purpose. This work can be further improved by optimizing and modifying the synthesis of MoS_2_ to develop uniform MoS_2_ single layer with the insertion of butyllithium by applying the pressure or temperature.

## 4. Conclusions

In this study, we successfully prepared MoS_2_ NS with Ag NP decoration, using the assistance of MPA functionalization. The structure and morphology of the Ag NPs on the MoS_2_ NSs were confirmed by SEM, XRD, and TEM measurements. The size of the MoS_2_ NSs was from 100 nm to ~3 μm. Ag NPs with a size of a few nm were decorated on the surface of the MoS_2_ NSs. The MoS_2_ NS shows inferior cycling performance of lithium storage capacity (~500 mAh·g^−1^) and rate performance. By incorporating Ag NPs, the storage capacity and rate performance of anodes were significantly improved. Among the three anodes prepared, the Ag3@MoS_2_ anode demonstrated the best cycling performance retention capacity of 73% compared to that in the first cycle after 100 cycles. Moreover, this anode could restore ~100% of the capacity after high rate performance. These results suggest that Ag-decorated MoS_2_ can be a potential anode for a high-rate and high-stability anode in lithium storage applications in the future.

## Figures and Tables

**Table 1:** Recent research of MoS_2_ nanosheets (NSs) for Li-ion storage.

Materials	Method	Phase of MoS_2_	Reversible Capacity after 100 Cycles at 0.1 A·g^−1^ (mAh·g^−1^)	Reference
Ag/MoS_2_ nanohybrids	Sonication	2H	~920 (after 50 cycles)	[24]
Sn/MoS_2_ composite	Hydrothermal	-	~1087	[43]
MoS_2_/reduced graphene oxide	Hydrothermal	-	~667	[44]
TiO_2_ decorated MoS_2_	Hydrothermal	2H	~604	[23]
Fe_2_O_3_@Carbon nanofiber/MoS_2_	Electrospinning and hydrothermal	2H	~900 (at 0.2 Ah·g^−1^)	[45]
1T MoS_2_	Liquid exfoliation assisted lithium molten salt at 1050 °C	1T	~855	[25]
Ag nanoparticles-decorated MoS_2_ NSs	Liquid exfoliation method	1T	~510	[This work]

## Data Availability

Data is contained within the article.
